# Accuracy of liquid-based brush cytology and HPV detection for the diagnosis and management of patients with oropharyngeal and oral cancer

**DOI:** 10.1007/s00784-021-04228-5

**Published:** 2021-11-27

**Authors:** Paola Castillo, Jorge de la Oliva, Silvia Alos, Francisco Perez, Naiara Vega, Isabel Vilaseca, Carles Marti, Ada Ferrer, Llucia Alos

**Affiliations:** 1 Department of Pathology, Hospital Clínic, Villarroel, 170, 08036 Barcelona, Spain; 2 School of Medicine, Universitat de Barcelona, Barcelona, Spain; 3 Institut dInvestigacions Biomèdiques August Pi I Sunyer, IDIBAPS, Barcelona, Spain; 4 Department of Otorhinolaryngology, Hospital Clínic, Barcelona, Spain; 5 Department of Maxillofacial Surgery, Hospital Clínic, Barcelona, Spain; 6 Head Neck Clínic, Agència de Gestió d’Ajuts Universitaris I de Recerca, 2017-SGR-01581 Barcelona, Spain

**Keywords:** High-risk human papillomavirus, Cytology cytobrush, Squamous cell carcinoma, p16, Oropharyngeal carcinoma, Oral carcinoma

## Abstract

**Objectives:**

This study aims to evaluate the usefulness of liquid-based brush cytology for malignancy diagnosis and HPV detection in patients with suspected oropharyngeal and oral carcinomas, as well as for the diagnosis of tumoral persistence after treatment.

**Material and methods:**

Seventy-five patients with suspicion of squamous cell carcinoma of the oropharynx or oral cavity were included. Two different study groups were analyzed according to the date of the sample collection: (1) during the first endoscopy exploration and (2) in the first control endoscopy after treatment for squamous cell carcinoma. Sensitivity, specificity, positive predictive value, negative predictive value, and accuracy for malignancy diagnosis as well as for HPV-DNA detection on brush cytologies were assessed.

**Results:**

Before treatment, the brush cytology showed a sensitivity of 88%, specificity of 100%, and accuracy of 88%. After treatment, it showed a sensitivity of 71%, specificity of 77%, and accuracy of 75%. HPV-DNA detection in cytology samples showed a sensitivity of 85%, specificity of 100%, and accuracy of 91% before treatment and an accuracy of 100% after treatment.

**Conclusions:**

Liquid-based brush cytology showed good accuracy for diagnosis of oropharyngeal and oral squamous cell carcinoma before treatment, but its value decreases after treatment. Nevertheless, it is useful for HPV-DNA detection, as well as to monitor the patients after treatment.

**Clinical relevance:**

Brush cytology samples are reliable for the detection of HPV-DNA before and after treatment and may be a useful method to incorporate in the HPV testing guidelines.

## Introduction

High-risk human papillomavirus (HPV) infection is a well-established risk factor for a proportion of head and neck squamous cell carcinomas, mostly located in the oropharynx [1, 2], with HPV type 16 (HPV-16) being the most frequently identified HPV genotype [3, 4].

The HPV-related oropharyngeal squamous cell carcinoma (OPSCC) has shown distinctive biology with different epidemiological, clinical, and prognostic features from HPV negative OPSCC. Particularly important are the improved response to treatment and survival of HPV-related tumors over the HPV negative tumors [5, 6] that lead to ongoing clinical trials of de-escalation treatments and aim to achieve good results with fewer treatment-associated comorbidities. Despite this better prognosis, around 10 to 25% of patients will suffer a disease recurrence within 5 years of treatment and another portion of patients will die from the disease [5, 7].

Thus, the determination of the HPV status is crucial to discriminate between HPV-related tumors from those which are not. Indeed, the HPV status is required in the American Joint Committee on Cancer (AJCC) 8th edition for the TNM of OPSCC staging classification [8]. For this reason, adequate detection methods for the establishment of HPV infection in head and neck carcinomas have been investigated in recent years [9–12].

The detection of HPV is based on immunohistochemical p16 expression as a surrogate marker for transcriptionally active high-risk HPV, HPV-DNA detection by in situ hybridization (ISH), HPV E6/E7 mRNA by ISH, type-specific polymerase chain reaction (PCR) techniques, and real-time PCR assays to quantify viral load [13, 14]. All these techniques are usually performed on the tumor sample biopsy or surgical specimen. However, interest in cytologic sampling is increasing due to its non-invasive, time-effective, and low-cost nature. Few studies have demonstrated the reliability and feasibility of liquid-based brush cytology specimens from oropharyngeal and oral lesions [10, 15, 16].

Patients’ management might benefit from cytology procedures not only for early diagnosis purposes but also as a follow-up diagnostic tool. This study aims to evaluate the usefulness of liquid-based brush cytology diagnosis and HPV detection for (1) the diagnosis of clinically suspected oropharyngeal and oral carcinomas and (2) the diagnosis of tumoral persistence after treatment for oropharyngeal or oral carcinomas.

## Material and methods

### Patients

**Fig. 1:**
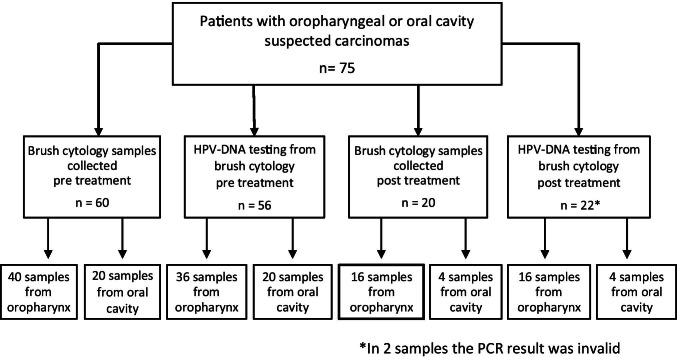
Number of cytobrush samples included in each study groups for cytology diagnosis and HPV-DNA testing

This cohort study was conducted at the Pathology Department of the Hospital Clínic of Barcelona, Spain. Seventy-five patients, first diagnosed or suspected of a squamous cell carcinoma of the oropharynx or oral cavity, were included in the study between 2015 and 2018 (Fig. 1). In the Otorhinolaryngology and/or Maxillofacial Departments, brush cytology was performed and two different study groups were analyzed according to the date of the sample collection: (1) samples collected during the first endoscopy examination before treatment and (2) samples collected from the tumor site in the first control endoscopy, after treatment for squamous cell carcinoma.

After treatment, a paired cytology and biopsy were collected in lesions with high suspicion of carcinoma, whereas lesions with low suspicion were studied only by cytology.

Informed consent was signed from all patients, and the study was approved by the local ethics committee.

### Brush cytology samples and HPV detection

In all cases, cytobrush heads were rotated on the lesional surface several times and transferred into a methanol-based preservative solution (ThinPrep Solution, Hologic Suisse, Lausanne, Switzerland). An aliquot of each sample was processed with liquid-based technology (ThinPrep 5000 Processor, Hologic), stained using the Papanicolaou method, and assessed under microscopy. All cases were revised by two pathologists (LA, JO) and one cytotechnologist (SA, FP, or NV) blinded to the clinical condition of the patient. Positive cytology was defined as the presence of atypical cells consistent with squamous cell carcinoma.

From another aliquot of each sample, HPV detection was performed using the Cobas HPV test (Roche Diagnostics), following published procedures [17]. This test is able to detect HPV 16, 18 and consensus high-risk HPV (31, 33, 35, 39, 45, 51, 52, 56, 58, 66, 68). β-globin was used as a housekeeping gene. Internal controls (HPV 16, 18, high-risk consensus, and β-globin plasmids) were used on each plate. PCR consisted of 50 cycles (93 °C and 56 °C) followed by 30 s of cooldown to 40 °C and 10 s at 25 °C. Data were finally assessed using LightCycler 480 SW 1.5.0 software (Roche Diagnostics).

### Histopathological analysis, p16 immunostain, and HPV detection on tissue samples

Biopsy of suspected lesions was performed in 60 patients. The biopsies were fixed in neutral-buffered formalin (4%) and processed for histological analysis using standard methods. Histologic diagnosis was rendered in each case from the hematoxylin and eosin–stained tissue section, according to the World Health Organization classification for head and neck tumors [18] and revised by two pathologists (LA, PC).

p16 staining was performed from formalin-fixed and paraffin-embedded tissue sections measuring 3 µm in thickness which were deparaffinized following antigen retrieval (ethylenediaminetetraacetic acid [pH 9.0] at 958C for 30 min). p16 staining (clone E6H4 [dilution 1:10] for 30 min; MTM Laboratories, Heidelberg, Germany) was performed on a Leica BOND MAX instrument (Leica Biosystems, Nussloch, Germany) using the Bond Polymer Refine Detection Kit (Leica). p16 positivity was defined as ≥ 70% tumor cells with strong nuclear and cytoplasmic staining [14].

Specifications on DNA extraction and HPV detection and typing are described elsewhere [19]. Briefly, HPV DNA amplification was performed using INNO-LiPA HPV Genotyping Extra II assay (INNO-LiPA; Fujirebio Europe, Ghent, Belgium). This probe assay allows the SPF10 consensus primers to amplify a 65 bp fragment of the L1 region of the HPV genome, followed by reverse line blot hybridization to HPV type-specific immobilized probes for 32 high-risk/possibly high-risk (16, 18, 25, 31, 33, 35, 39, 45, 51, 52, 53, 56, 58, 59, 66, 68, 73, and 82) and 7 low-risk (6, 11, 40, 43, 44, 54, and 70).

In all lesions, the HPV status was assessed following the established guidelines [18]. p16 was performed systematically in all samples from the oropharynx. In non-keratinizing oropharyngeal SCC, p16 positivity was considered a surrogate for transcriptionally active high-risk HPV [18]. HPV detection in tissue was performed in discordant cases between the biopsy results and the HPV-DNA detection on cytology samples. In the oral cavity, outside the oropharynx, keratinizing SCCs were considered non-associated HPV carcinomas [18].

### Statistics

Data were analyzed using IBM SPSS for windows version 24.0. A *p*-value of 0.05 was defined as statistically significant. To evaluate sensitivity, specificity, positive/negative predictive value, and accuracy of cytology test results after treatment, the disease was considered present (SCC persistence) when resulting in a confirmed positive biopsy, whereas no malignancy (absence of SCC persistence) was assessed by the follow-up of patients who did not show tumoral persistence or recurrence in the next 12 months.

## Results

### Study cohort

From the 75 patients with a suspected carcinoma, a squamous cell carcinoma (SCC) was diagnosed in 73 cases (97%), 72 of which were infiltrative SCC, one with in situ SCC. The remaining 2 lesions (3%) were diagnosed with oropharynx lymphoid hyperplasia.

Figure 1 shows the number of cytobrush samples included in each study group for cytology diagnosis and HPV-DNA testing.

The demographic characteristics and tumor stage of all patients diagnosed with squamous cell carcinoma included in the study are presented in Table 1.

**Table 1:** Clinicopathological features of patients diagnosed with squamous cell carcinoma (*n* = 73) included in the study

	Oropharyngeal carcinomas	Oral cavity carcinomas
Total	49 (67.1%)	24 (32.9%)
Sex		
Male	39 (79.6%)	14 (58.3%)
Female	10 (20.4%)	10 (41.6%)
Age (median (range))	63 (42–92)	61 (47–87)
Tobacco smoke	34 (69.4%)	16 (66.7%)
Alcohol abuse	24 (48.9%)	8 (33.3%)
Stage of tumors		
0 (Tis)	0	1 (4.3%)
I	3 (6.1%)	2 (8.7%)
II	7 (14.3%)	3 (13.0%)
III	10 (20.4%)	2 (8.7%)
IV	28 (59.2%)	16 (65.2%)

### Value of the liquid-based brush cytology for the diagnosis of oropharyngeal and oral lesions before treatment

Paired biopsy and cytology analysis were obtained pre-treatment in 60 samples from the oropharynx (*n* = 40) and oral cavity (*n* = 20). The test showed high sensitivity and specificity (88% and 100%, respectively) and high accuracy (88%) for the diagnosis of squamous cell carcinoma (Table 2). Disagreement between the cytology and the biopsy was considered in 5 cases where the cytology material was scarce, insufficient for diagnosis, and in 2 cases described as negative for malignancy (a total of 7 false-negative cases).

**Table 2:** Value of the cytology sample for the diagnosis of oropharyngeal and oral cavity lesions before treatment. Sensitivity, specificity, positive, negative predictive value (PPV and NPV), and accuracy

Biopsy diagnosis	Total cases	Cytology diagnosis	
		Positive (SCC)	Negative
SCC	58	51	7
Lymphoid hyperplasia	2	0	2
Total	60	51	9

Sensitivity: 88%; specificity: 100%; PPV: 100%; NPV: 22%; accuracy: 88%

*SCC* squamous cell carcinoma

### Value of cytology samples for the HPV detection in oropharyngeal and oral lesions before treatment

The HPV-DNA detection on cytology samples was determined in 56 samples, from the oropharynx (*n* = 36) and oral cavity (*n* = 20). The samples from the oropharynx included the 2 lymphoid hyperplasia that were negative for HPV-DNA and 34 SCC. On these SCCs, HPV-DNA positivity was detected in 11 cytobrush samples (11/34; 32%), identifying an HPV16 genotype in all but one sample. A discrepancy was observed in two non-keratinizing SCCs (2/34; 6%), in which the p16 immunostain in the tissue sample was positive, whereas the HPV-DNA detection in the cytobrush sample was negative. In both cases, HPV-DNA testing was re-conducted in the tissue samples showing positivity for HPV16 in the further analysis. Thus, the HPV detection using cytobrush samples from oropharyngeal squamous cell carcinoma showed 85% sensitivity, 100% specificity, and 91% accuracy (Table 3).

**Table 3:** Value of the HPV detection on cytology samples from oropharyngeal squamous cell carcinomas before treatment. Sensitivity, specificity, positive, negative predictive value (PPV and NPV), and accuracy

HPV status according to the biopsy sample *	Total cases	Cytology HPV detection	
		HPV +	HPV −
Positive	13	11	2
Negative	21	0	21
Total	34	11	23

Sensitivity: 85%; specificity: 100%; PPV: 100%; NPV: 91%; accuracy: 91%

*HPV* human papilloma virus

^*^HPV status: HPV-associated squamous cell carcinoma (SCC) showed non-keratinizing morphology and p16 positive immunostain. In the two discordant cases with the HPV-DNA detection on cytology, HPV positivity was confirmed with HPV-DNA detection in tissue

All 20 squamous cell carcinomas from the oral cavity showed HPV-DNA negativity, according to a keratinizing SCC morphology and location outside the oropharynx.

### Value of the liquid-based brush cytology for the diagnosis of oropharyngeal and oral carcinoma persistence after treatment

During the follow-up after treatment, brush cytology was assessed in a total of 20 samples from the oropharynx (*n* = 16) and oral cavity (*n* = 4) tumor site. Patients had been treated with surgery (*n* = 3), radiotherapy alone (*n* = 4), and/or a combination with chemotherapy (*n* = 13). The samples were collected from 36 to 249 (median 136) days after finishing treatment.

**Fig. 2:**
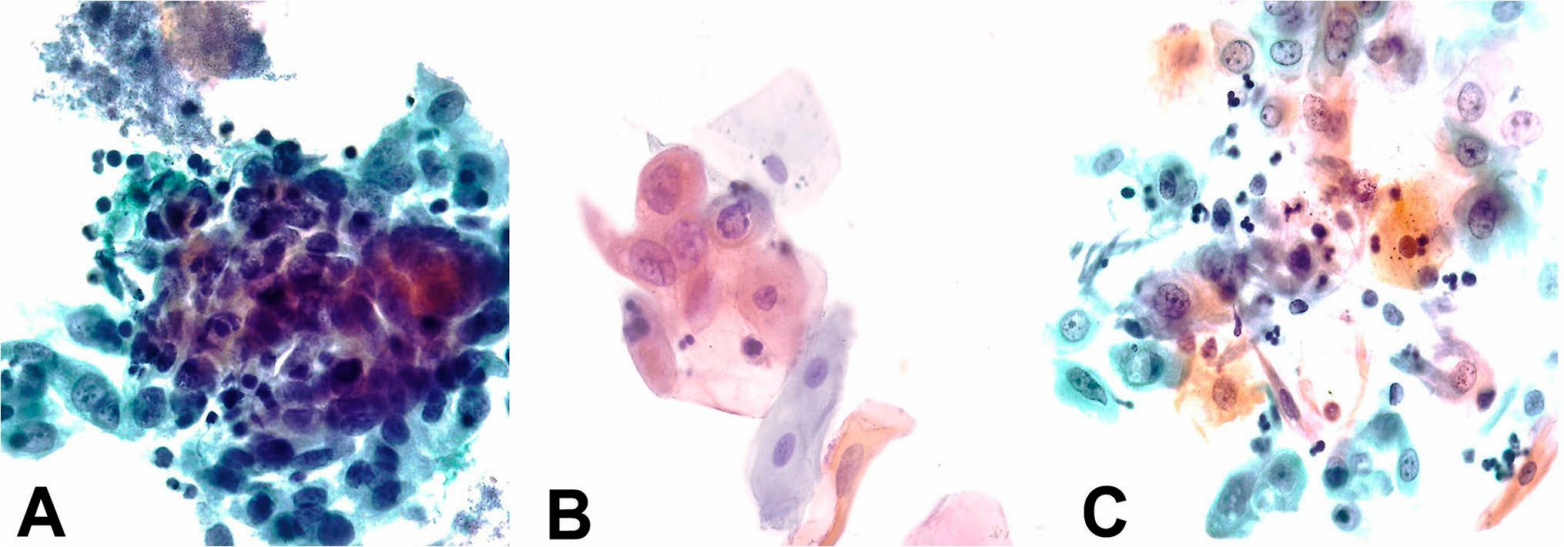
Examples of cytologic specimens from oropharyngeal and oral cavity squamous cell carcinomas: **a** atypical cells from an oropharyngeal HPV-related squamous cell carcinoma, before treatment; **b** atypical cells from an oral cavity squamous cell carcinoma, before treatment; **c** atypical cells from the oropharynx in a patient without tumoral persistence, attributed to reactive changes secondary to received treatment. Note the similarities within the three samples, all showing high nucleus/cytoplasm ratio, visible and irregularly dispersed nucleoli, and atypical chromatin with nuclear clearing. Elongated, spindle-shaped cells are observed in samples B and C

The lesions were studied by cytology and biopsy simultaneously in 9 cases showing high clinical suspicion for carcinoma and only cytology in 11 showing low suspicion of malignancy. The overall results of the cytology after treatment (patients with concomitant biopsy or long follow-up) showed a sensitivity of 71.4%, specificity of 76.9%, and accuracy of 75% (Table 4). The discrepancies were observed in 5 cases: 2 false-negative results for malignancy and 3 false-positive results that showed atypical cells attributed to post-treatment reactive changes (Fig. 2).

**Table 4:** Value of the cytology sample for the diagnosis of oropharyngeal and oral cavity lesions after treatment. Sensitivity, specificity, positive, negative predictive value (PPV and NPV), and accuracy

Diagnosis	Total cases	Cytology diagnosis	
		Positive (SCC)	Negative
SCC persistence	7	5	2
No malignancy	13	3	10
Total	20	8	12

Sensitivity: 71.4%; specificity: 76.9%; PPV: 63%; NPV: 83%; accuracy: 75%

*SCC* squamous cell carcinoma

SCC persistence: squamous cell carcinoma persistence was confirmed by positive biopsies. No malignancy: the absence of SCC was assessed by the follow-up of patients who did not show tumoral persistence or recurrence

### Value of cytology samples for the HPV detection in oropharyngeal and oral sites after treatment

HPV-DNA detection was available in 22 cytobrushes performed after treatment, demonstrating 19 HPV-DNA negative cases, and one case HPV16-DNA positive. The HPV-positive case corresponded to an HPV-related SCC treated with chemotherapy and radiotherapy with tumoral persistence 3 months after treatment, confirmed by biopsy and cytology.

The 19 HPV negative results were consistent with the HPV expected status after treatment: 8 oropharynx HPV-associated SCC with no tumoral persistence after treatment and 11 SCC that were HPV-non associated SCC before treatment (7 oropharynx SCC and 4 oral cavity SCC). Thus, the HPV detection using cytobrush samples showed 100% sensitivity, specificity, and accuracy (Table 5).

**Table 5:** Value of the HPV detection on cytology samples from oropharyngeal and oral cavity lesions after treatment. Sensitivity, specificity, positive, negative predictive value (PPV and NPV), and accuracy

HPV expected status after treatment *	Total cases	Cytology	HPV detection
		HPV +	HPV −
HPV positive	1	1	0
HPV negative	19	0	19
Total	20	1	19

Sensitivity: 100%; specificity: 100%; PPV: 100%; NPV: 100%; accuracy: 100%

*HPV* human papilloma virus

^*^HPV expected status: HPV positive: SCC-HPV-associated persistence. HPV negative: SCC-HPV-associated without SCC persistence and non-associated HPV SCC before treatment

In two cases (2/22; 9%), the cytobrush sample had insufficient DNA quality or quantity for testing, leading to invalid results.

## Discussion

To the best of our knowledge, this is the first study to assess the usefulness of both liquid-based cytology and HPV detection in patients with oropharyngeal and oral cancer before and after treatment. Only Kofler et al. have also evaluated post-treatment samples but only from the oropharynx and for HPV determination [20].

According to our results, the study of morphology on liquid-based cytology obtained by cytobrush before and after treatment may give equivocal results for squamous cell carcinoma diagnosis, although it can be of great utility for the detection of HPV-DNA.

The use of cytobrush for further liquid-based cytology is a well-tolerated and minimally invasive technique for collecting samples [16, 21]; however, a systematic review by Alsarraf et al. indicates variable results without clear recommendations on the effectiveness of this technique for the diagnosis of lesions on the head and neck [22]. In this regard, our cytology results from samples performed before treatment, when compared with the biopsy (gold standard), showed a high sensitivity (over 80%) and specificity (100%). The sensitivity results are in line with published data by Donà et al., with 164 patients who showed a sensitivity of 75.5%, although showing a low specificity. The high specificity observed in our series might be related to the chosen methodology for the cytomorphology evaluation. In our study, the presence of “atypical cells” was classified as positive for malignancy. A larger cohort, although limited to oral samples, has shown a specificity rising to 84.9% [21], also similar to our results. A plausible explanation for the false-negative results found in our study could be the inaccessibility of the lesions. Anatomical limitations particularly in the oropharynx, in which SCC can develop in the tonsillar crypts, are likely a limiting factor in sampling with cytobrush [23, 24].

In addition, we evaluated the value of the cytomorphology for the diagnosis after treatment. To that purpose, the cytology results were evaluated against the persistence or not of SCC, assessed by biopsy or by the follow-up of the patients. To our understanding, to date, no data is rendered on the effectiveness of this technique in samples taken post-treatment since most authors center on the cytology utility as a screening test or for initial diagnosis and not for follow-up [22, 25]. Our results indicate a moderate accuracy (75%), sensitivity (71.4%), and specificity (76.9%) for cytology diagnosis in these samples. Its low accuracy makes this technique less effective when used alone to monitor patients after treatment. The false-positive cases observed in our study showed “atypical” cells, most likely an overinterpretation of reparative changes as a consequence of the treatment received, representing a barrier when evaluating cytomorphology. Moreover, cytologic abnormalities in oropharyngeal brushings and oral rinses can be associated with smoking and drinking habits [22, 26].

Concerning HPV detection, our results correlate with several studies confirming that cytobrush samples are useful in determining HPV status with the Roche Cobas HPV test [7, 27, 28]. Before treatment, samples were evaluated against the p16 immunostain or tissue HPV-DNA detection. For this reason, the analysis was done only in oropharyngeal samples where the immunostain was done routinely as a surrogate marker. However, the absence of HPV detection in all oral SCC tested correlates with the keratinizing morphology and location outside the oropharynx, following the established clinical guides and our experience [18, 29]. Our results support the HPV detection in cytobrush samples as a valid and reliable alternative method for the assessment of HPV in oropharyngeal and oral carcinoma. Access to easy but robust methods is particularly necessary, with increasing data indicating the need for double testing for HPV since double positivity for HPV-DNA/p16INK4a showed the strongest diagnostic accuracy and prognostic value [30]. The stratification of the patients could rise in importance as de-escalation strategies will be finally established. Although several ongoing clinical trials are aiming to reduce treatment-associated toxicities without affecting the superior survival rates, there is still a lack of strong evidence to currently recommend any of the chemotherapy schemes proposed for de-escalation treatment [31]. 

In our study, erroneous stratification would be performed if HPV status was based in one method alone. In 2 HPV-associated oropharyngeal carcinomas (6%), HPV-DNA was not detected in the cytology sample. In addition, the HPV status based only on the p16 immunostain could falsely classify the patients as HPV positive or negative, according to the results in the literature, ranging from 4.9 to 26.2% [32, 33]

The validation of methods for the correct diagnosis of HPV status in post-treated patients is crucial for prognosis and surveillance. Our results showed that HPV determination in samples after treatment successfully correlates with the HPV expected status post-treatment with an accuracy of 100%. In our series, only one patient with HPV16-related tumor showed tumoral persistence, which correlates with the HPV-DNA positivity in the post-treatment sample. As shown in the data published by Kofler et al., the same genotype as that identified in the pre-treatment analysis was identified in the HPV-positive post-treatment sample. In our study, HPV-associated OPSCC with an HPV negative result after treatment correlated with no tumoral persistence, in agreement with Kofler et al. results [20]. Nevertheless, we have to take into account that in up to 9% of the cohort the test was invalid, attributed to the scarce number of cells and insufficient DNA detection in these samples.

Importantly, the selection of cytobrush procedure instead of using oral rinses for the extraction of the samples might play a part in the good results observed in our study. Some previous studies have compared the performance of cytobrush samples vs oral rinses samples for HPV determination and found poor agreement between the two methods [34] and stated that oral rinse cytology showed lower sensitivity (43–88%) for the assessment of HPV infection [35]. Indeed, technical problems were identified during the study as scarce cells were identified in some cases which led to false-negative results. To overcome this technical issue, the collection of the samples should be obtained by performing several passes of the cytobrush in the suspected area. Another limitation of our study includes the sample size, particularly in the group of patients with paired biopsy and cytology after treatment.

Due to ethical issues, patients with low suspicion of carcinoma persistence were not biopsied. However, a long follow-up of the patients confirmed the absence of tumoral persistence after treatment in this group of patients. In addition, in our series, only squamous cell carcinomas were evaluated, so the render of cytology in dysplastic lesions was not assessed in our study. To properly address cytology accuracy for diagnosis, the tested samples should comprise more negative samples.

All results considered, this study contributes to raising the evidence on the effectiveness of cytobrush for establishing the HPV status in oral and oropharyngeal carcinomas, which could improve the diagnostic approaches and algorithms. This study also confirms the usefulness of these samples to rule out HPV-associated oropharyngeal carcinoma persistence after treatment, as a potential method to monitor the patients.

## Conclusions

Brush cytology samples are reliable for the detection of HPV DNA before and after treatment and may be a useful method to incorporate in the HPV testing guidelines. Brush cytology morphology showed fair results for the diagnosis of SCC before and after treatment. Further analysis on a larger patient cohort is necessary to confirm our results.
